# Cardiac arrest caused by acute gastric dilatation in a bulimia patient: a case report

**DOI:** 10.3389/fmed.2026.1738665

**Published:** 2026-03-13

**Authors:** Qi Cai, Menglong Song, Hongqiong Peng, Hua Jiang, Ping Zhou

**Affiliations:** 1 School of Medicine, University of Electronic Science and Technology of China, Chengdu, China; 2 Department of Emergency Medicine, School of Medicine, Sichuan Provincial People's Hospital, University of Electronic Science and Technology of China, Chengdu, China

**Keywords:** bulimia nervosa, Acute gastric dilatation, Cardiac arrest, Extracorporeal membrane oxygenation (ECMO), Surgical delay

## Abstract

This case report describes a young female patient with a long-standing history of bulimia nervosa a who developed acute gastric dilatation (AGD) following a binge-eating episode. The patient declined surgical intervention, leading to rapid clinical deterioration and cardiac arrest. Despite achieving return of spontaneous circulation (ROSC) after cardiopulmonary resuscitation (CPR), followed by emergency surgery and extracorporeal membrane oxygenation (ECMO) support, she remained hemodynamically unstable and eventually died. This case highlights the critical severity of AGD and underscores the importance of early surgical intervention, while also discussing the limitations of ECMO in managing non-cardiogenic shock.

## Introduction

Acute gastric dilatation (AGD) was first described by S. E. Dupuy in 1833 as a rare but severe clinical condition (1). The disease progresses rapidly, typically within 24 h after the onset of acute gastric distension. If not promptly recognized and treated, it can lead to gastric ischemic necrosis and perforation. Epidemiological data indicate that AGD occurs more frequently in females (67%) and most commonly involves the gastric curvature (63%). If this situation occurs without timely surgical intervention, it can lead to death, with an overall mortality rate as high as 73% (2, 3). Extracorporeal membrane oxygenation (ECMO) is an advanced life-support technique that provides temporary circulatory and/or respiratory assistance but does not address the underlying disease or injury. It has significantly improved survival in critically ill patients who fail to respond to conventional life-support measures (4). Depending on the reinfusion pathway, ECMO is classified into two main modalities: veno-venous (VV) and veno-arterial (VA). VA-ECMO primarily supports circulation in patients with cardiac failure, whereas VV-ECMO is preferred for those with isolated respiratory failure (2, 5, 6). VA-ECMO serves as a potential rescue therapy for refractory cardiogenic shock and cardiac arrest because it can rapidly restore hemodynamic stability and gas exchange as a temporary mechanical circulatory support system (7). However, its role in managing non-cardiogenic shock remains controversial.

## Case description

A 26-year-old woman (height, 156 cm; weight, 47 kg) presented to the Gastroenterology Clinic of Sichuan Provincial People’s Hospital on September 12, 2024, at 15:00 with abdominal distension and pain following excessive food intake. On examination, she was conscious, with a heart rate of 150 bpm, respiratory rate of 32 breaths/min, and blood pressure of 114/68 mmHg. Marked abdominal distension was noted, accompanied by diffuse tenderness, mild rebound tenderness, and decreased bowel sounds. Abdominal CT revealed massive gastric distension with abundant contents and gastroptosis extending to the pelvic level. Based on these findings, a preliminary diagnosis of acute gastric dilatation (AGD) was made. She had a history of long-standing bulimia nervosa and had previously presented to our hospital on January 23, 2022, with postprandial abdominal distension. CT at that time also demonstrated marked gastric distension with abundant gastric contents and gastroptosis. At that time she improved with symptomatic conservative management.

**Figure 1:**
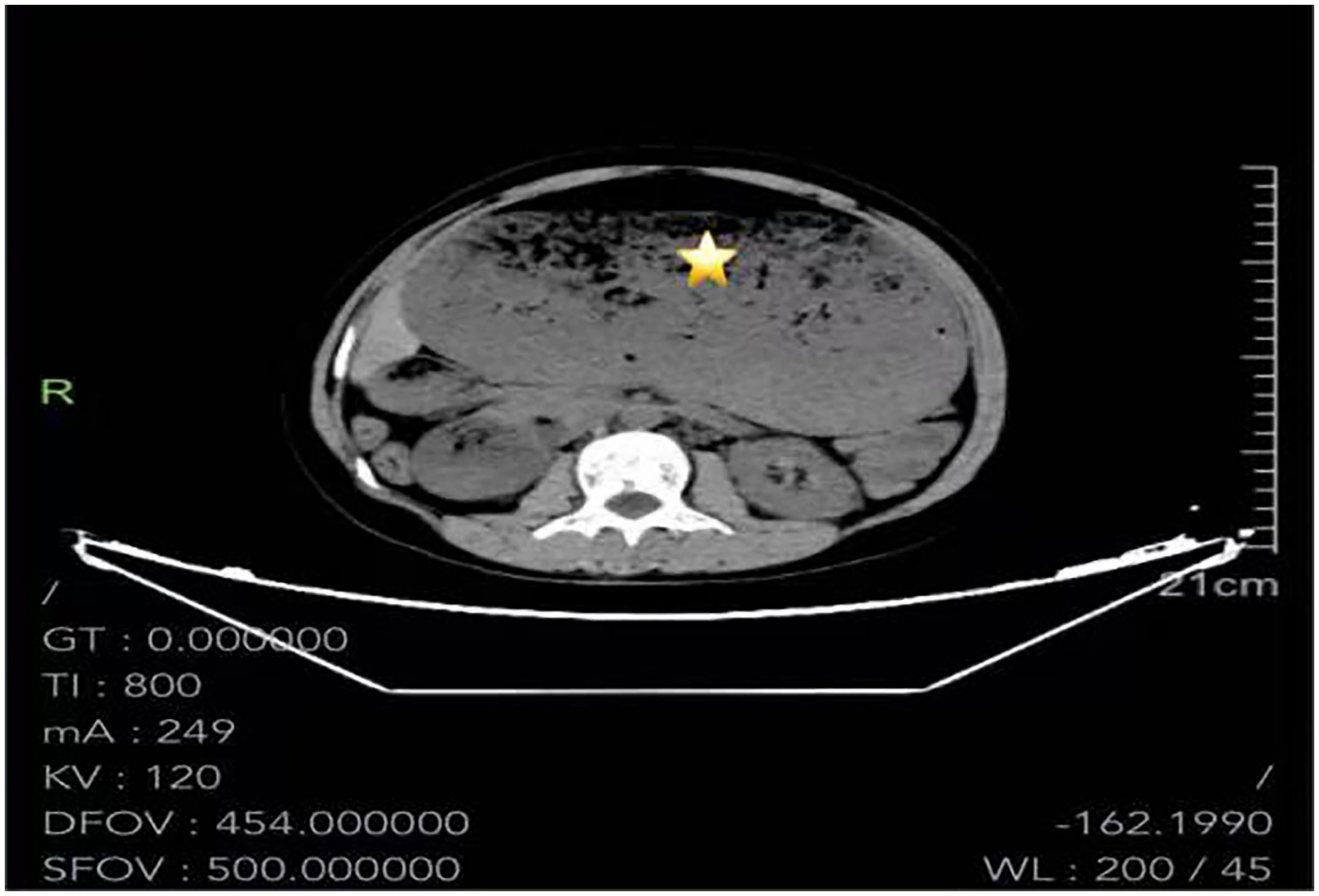
Abdominal CT scan shows that the enlarged stomach is filled with a large amount of gas and food residues.

Two hours later, the patient was admitted to the emergency observation unit. A Fr 16 nasogastric tube was inserted for gastric decompression, along with enemas and conservative supportive treatment. Eight hours later, the patient reported progressive worsening of abdominal pain and distension. An emergent abdominal CT scan performed at 02:02 on September 13, 2024. Figure 1 demonstrated marked dilatation of the stomach and duodenum with prominent air–fluid levels, a large volume of intragastric contents with patchy hyperdense areas, and compression of intra-abdominal structures. The nasogastric tube was noted to be refluxed into the distal esophagus. Following CT examination, the nasogastric tube was replaced with a larger-bore Fr 20 tube. Two hours later, the patient again developed worsening abdominal distension and respiratory distress. Vital signs showed a heart rate of 158 beats/min, respiratory rate of 22 breaths/min, and blood pressure of 125/85 mmHg. Under nasal cannula oxygen at 3 L/min, oxygen saturation was 95%. Arterial blood gas analysis revealed pH 7.36, PaCO₂ 24.4 mmHg, PaO₂147 mmHg, potassium 4.2 mmol/L, and lactate 6.8 mmol/L.

**Figure 2:**
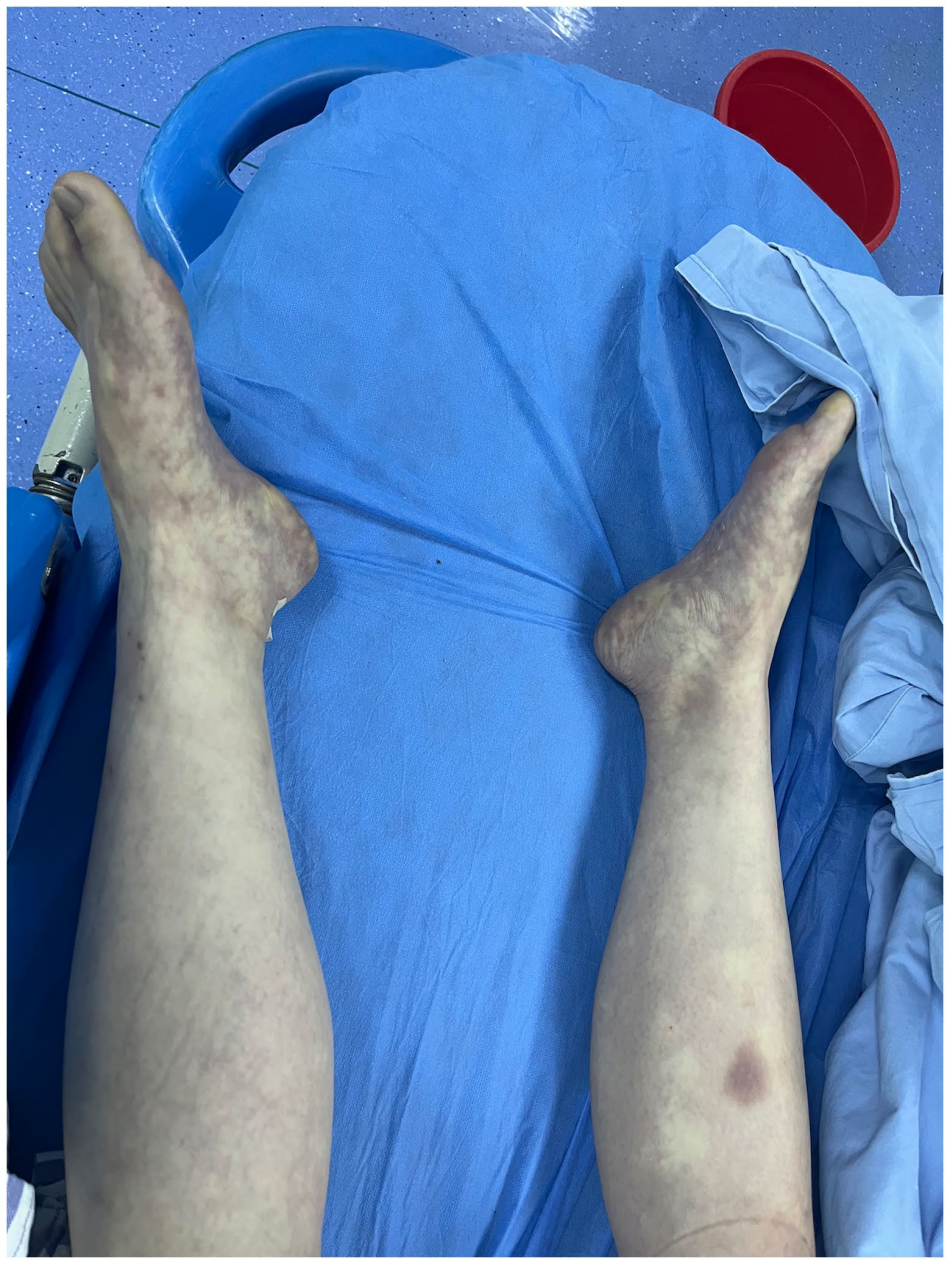
Scattered livid macules can be seen on both lower extremities.

At 07:51, emergency exploratory surgery was scheduled. Preoperative assessment revealed that she was conscious but tachypneic, with a heart rate of 154 bpm, respiratory rate of 20 breaths/min, and blood pressure of 70/45 mmHg. She exhibited severe abdominal distension with a rigid, board-like abdomen, tympanic percussion throughout, sensory impairment below the chest, and complete loss of muscle strength in the lower limbs. Her abdomen and body appeared mottled (Figure 2) and arterial blood gas showed a lactate level of 19 mmol/L, indicating severe metabolic acidosis and tissue hypoxia.

At 08:35, while still awake and before induction of anesthesia, she vomited a large volume of gastric contents. Afterward, she reported relief of symptoms and requested cancelation of surgery, signing a refusal consent form. At 08:50, the patient was conscious and experienced recurrent vomiting in the operating room, expelling a large volume of gastric contents, and again refused surgical intervention. At 09:05, while still conscious, the patient was assisted from the operating table to a transport stretcher in preparation for transfer to the ICU. At 09:08, the patient vomited for the third time, continued to refuse surgery, and shortly thereafter developed sudden cardiac and respiratory arrest.

Immediate multidisciplinary resuscitation was initiated, including bedside cardiopulmonary resuscitation, endotracheal intubation, intravenous administration of resuscitation medications, chest compressions, and defibrillation. At 09:18, the patient successfully achieved return of spontaneous circulation (ROSC), followed by thorough airway suctioning under fiberoptic bronchoscopy. At 10:08, after detailed communication with the patient’s family, informed consent was obtained, and emergency exploratory laparotomy with gastrotomy for removal of gastric contents was performed at 10:20. Intraoperatively, the patient developed recurrent refractory hypotension unresponsive to vasopressors, and repeated arterial blood gas analyses revealed persistent severe acidosis with pH values consistently below 7.0. The stomach was incised along the greater curvature, evacuated of contents, and closed, followed by abdominal closure. Operative time was 1 h 53 min. Findings revealed massive gastric dilatation extending into the pelvis, with a thin, edematous, partially darkened gastric wall. The stomach contained large amounts of foul-smelling, semi-digested food (notably mushrooms and kelp). Approximately 500 mL of yellow peritoneal exudate was also present.

Postoperatively, the patient was transferred to the emergency intensive care unit for continued management. Three hours after surgery, at 15:22, the patient’s heart rate began to decline rapidly and progressed to asystole. Immediate conventional cardiopulmonary resuscitation (CCPR) was initiated. After 15 min of high-quality CPR, sustained return of spontaneous circulation (ROSC) was not achieved. Bedside echocardiography demonstrated cardiac standstill with a left ventricular ejection fraction (LVEF) < 10%. Given refractory cardiac arrest, extracorporeal cardiopulmonary resuscitation (ECPR) was initiated as a salvage therapy. Approximately 30 min later, VA-ECMO was successfully established; however, ECMO flow remained unstable and could only be maintained at 1.0–1.5 L/min. During this period, continuous hemorrhagic drainage was observed from the abdominal drain, with a total output of approximately 4,200 mL, accompanied by active bleeding from multiple vascular access sites^.^ Laboratory findings revealed a rapid decline in hemoglobin from 141 g/L to 52 g/L and platelets from 276 × 10^9^/L to 31 × 10^9^/L. Anti–factor Xa activity (unfractionated heparin) was 0.1 IU/mL, with an activated clotting time (ACT) of 278 s. In addition, interleukin-6 levels were markedly elevated (25,000.62 pg./mL). Based on these findings and the clinical presentation, disseminated intravascular coagulation (DIC) was suspected, and a heparin-free anticoagulation strategy was adopted. Massive transfusion therapy was administered, including 17.5 units of packed red blood cells, 1 unit of platelets, 1,450 mL of fresh frozen plasma, and 10 units of cryoprecipitate, in an attempt to correct coagulopathy and hemorrhagic shock. Five hours after ECMO initiation, echocardiography demonstrated partial recovery of LVEF to 42.1%; however, stroke volume remained critically low (22.1 mL), with a left ventricular outflow tract velocity–time integral (VTI) of 10.4 cm. Cerebral oxygen saturation monitoring revealed a value of 28.4%. Throughout this period, serum lactate levels remained persistently elevated, fluctuating between 15 and 25 mmol/L. Despite aggressive transfusion, high-dose vasoactive support, and ECMO assistance, circulatory failure progressed. At 02:40 on September 14, 2024, ECMO flow could no longer be maintained, cardiac activity remained absent, and the patient was pronounced clinically deceased.

**Figure 3:**
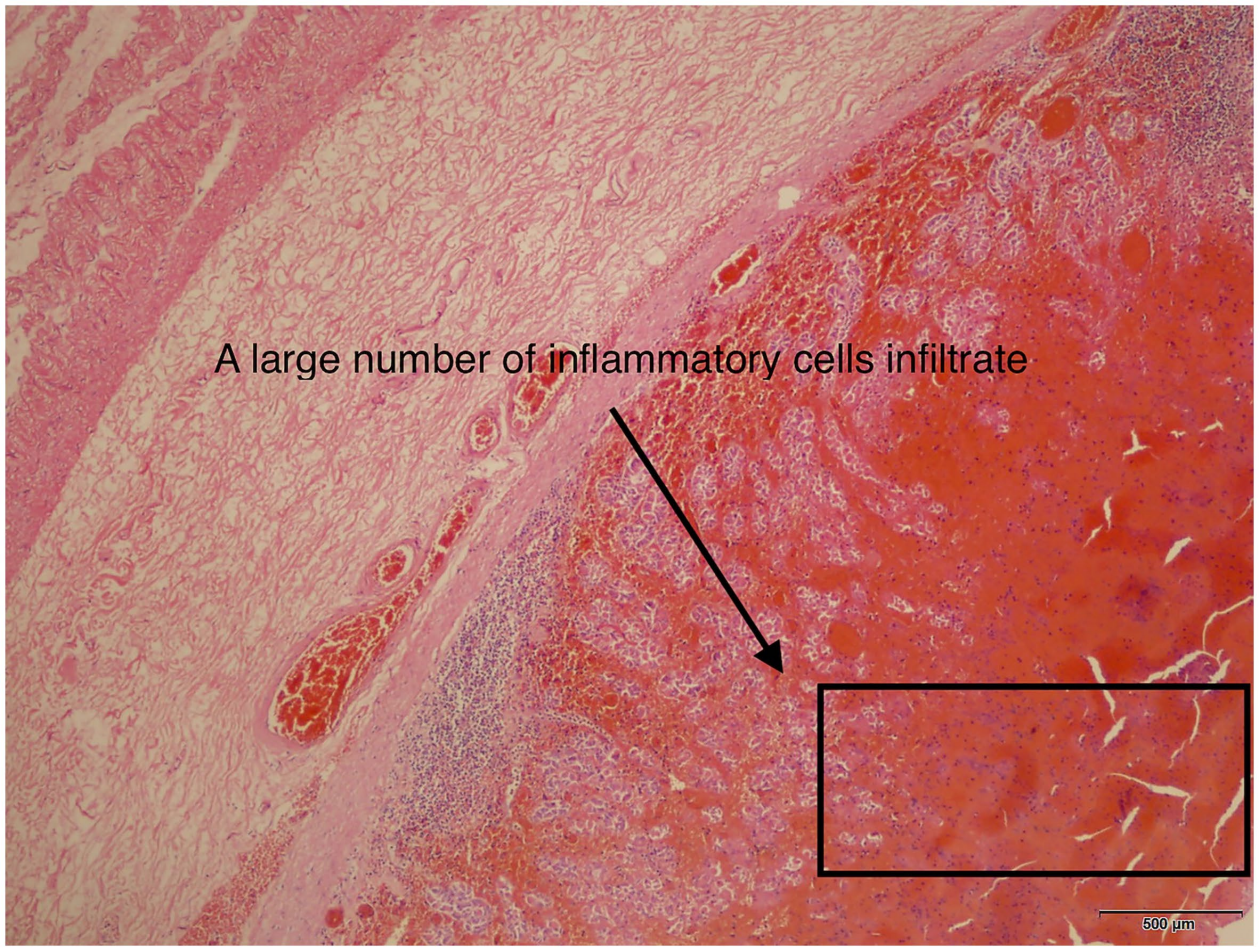
Under a 40-times magnification, the gastric pathological report shows a large amount of inflammatory exudate.

**Figure 4:**
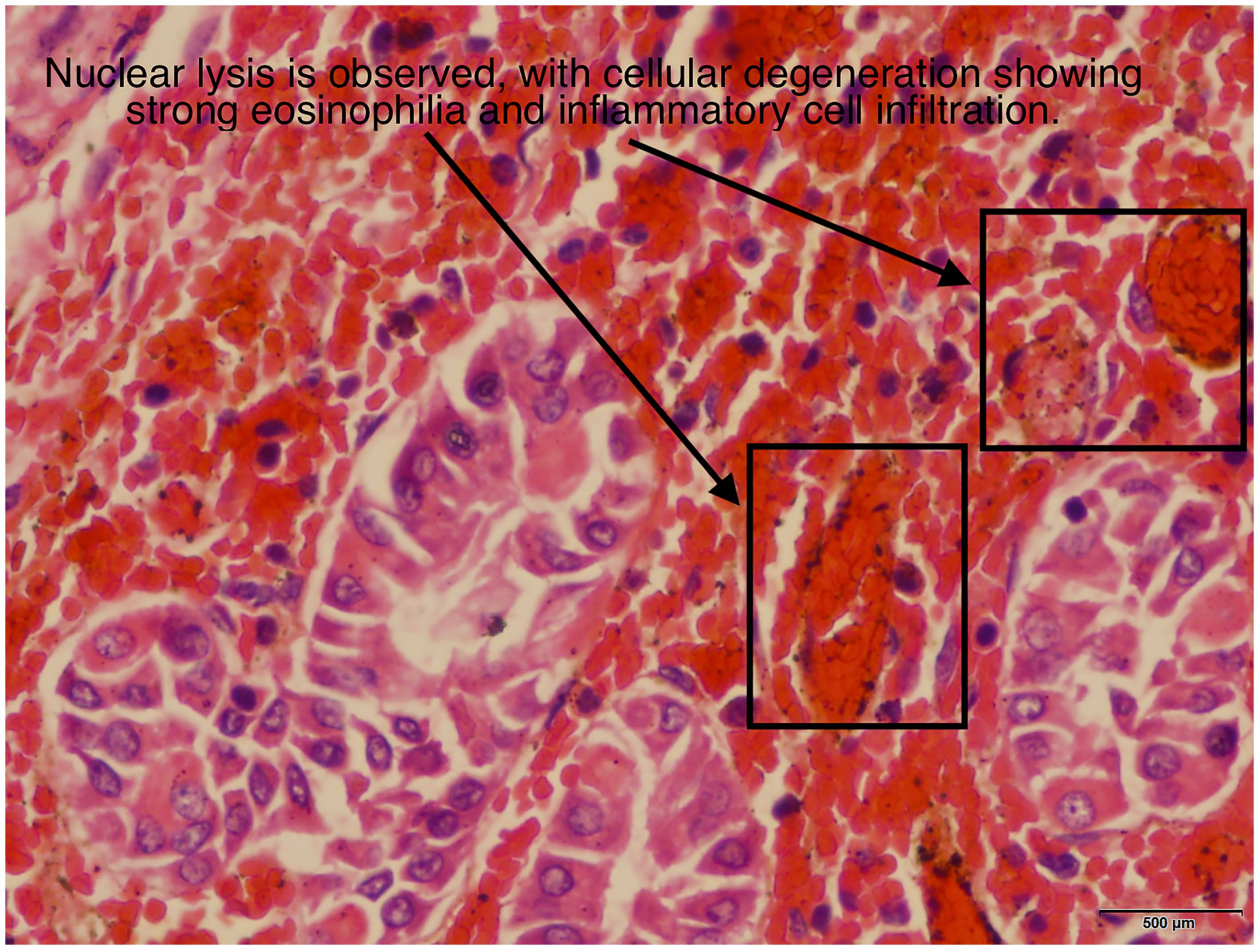
Under a magnification of 400 times, the gastric pathological report shows nuclear pyknosis and necrosis, and the cytoplasm is deformed with strong eosinophilia.

The postoperative pathological report indicated the following: “Gastric Posterior Wall”: The excised stomach appeared dark red and necrotic. Microscopic examination showed full-thickness hemorrhage and necrosis, with vascular dilation and congestion in the submucosa and serosa (Figures 3, 4).

## Discussion

Acute gastric dilatation (AGD) is a rare clinical condition characterized by diffuse abdominal pain, distension, nausea, and vomiting (8). More than 90% of patients with AGD experience vomiting (9). The etiology of AGD remains unclear and can be broadly classified into mechanical and non-mechanical causes. Mechanical causes include pyloric stenosis, gastric volvulus, superior mesenteric artery syndrome, and any abnormality that leads to complete gastric outlet obstruction. Non-mechanical causes include eating disorders such as anorexia nervosa and bulimia nervosa, autonomic neuropathy secondary to electrolyte imbalance or diabetes, as well as local infections, medications, or postoperative factors (10–12).

The diagnosis of AGD relies primarily on clinical presentation and imaging studies, such as abdominal X-ray and computed tomography (CT), which typically demonstrate marked gastric distension with large amounts of gas or fluid. Endoscopy may be useful for identifying the underlying cause of obstruction and evaluating ischemic mucosal changes; however, imaging should always be performed first to rule out perforation before endoscopy (13, 14). Several reports have emphasized that even in patients without a prior history of eating disorders, AGD should be considered in young women presenting with abdominal pain, nausea, or vomiting following a binge-eating episode (15).

Gastric ischemic necrosis and perforation are the most severe complications of AGD and are associated with extremely high mortality. Although the stomach is richly vascularized with extensive collateral circulation, once intragastric pressure exceeds 20–30 cm H₂O, gastric wall perfusion can be compromised, resulting in ischemia, necrosis, or even rupture (16). Previous case reports have shown that in patients with eating disorders, acute gastric dilatation following binge eating can result in gastric volumes as large as 15 liters (17)^.^

Management strategies for acute gastric dilatation (AGD) vary widely, ranging from conservative measures to invasive surgical interventions. In hemodynamically stable patients, nasogastric decompression is considered the first-line treatment (13), accompanied by thorough evaluation and management of underlying etiologies. However, delayed complications such as perforation or bleeding may still occur even after successful decompression. Following decompression, upper gastrointestinal endoscopy is often recommended to assess gastric mucosal integrity and facilitate aspiration of residual gastric contents (14). In cases of recurrent AGD or AGD secondary to acute gastric volvulus, therapeutic upper endoscopy may be employed for endoscopic reduction and decompression (18). Although gastric outlet obstruction itself may precipitate AGD, endoscopic pyloric stent placement using self-expanding metal stents is primarily indicated for mechanical obstruction, most commonly caused by advanced malignancy, and serves a palliative role by restoring luminal patency and improving nutritional intake (19). This approach has not been recommended for non-mechanical obstruction, such as functional gastroparesis (20). Importantly, in the present case, CT imaging demonstrated severe gastric wall ischemia and edema resulting from extreme dilatation. Under these pathological conditions, placement of a rigid stent would neither correct the underlying motility disorder nor provide effective decompression, but could instead increase the risk of iatrogenic gastric perforation or bleeding due to local pressure effects, potentially exacerbating the patient’s clinical course.

Although extracorporeal membrane oxygenation (ECMO) does not address the underlying cause of sepsis, such as the infectious source or the resulting dysregulated inflammatory response, it may serve as a temporary life-support strategy in selected patients with severe sepsis or septic shock (21). The core pathophysiological features of sepsis include dysregulated systemic inflammation, microcirculatory dysfunction, and impaired cellular oxygen utilization. When conventional therapies, including fluid resuscitation, vasoactive agents, and mechanical ventilation, fail to maintain adequate oxygenation and tissue perfusion, ECMO can provide extracorporeal gas exchange and circulatory support to transiently improve global oxygen delivery and hemodynamic stability (22). Two ECMO configurations are commonly employed in sepsis. Veno-venous ECMO (VV-ECMO) is primarily indicated for septic patients with severe acute respiratory distress syndrome, whereas veno-arterial ECMO (VA-ECMO) may be considered to temporarily support cardiac output and end-organ perfusion, thereby providing a therapeutic window for source control, antimicrobial therapy, and organ recovery in selected cases (23, 24).

Sepsis is characterized by heterogeneous hemodynamic phenotypes. The first is distributive shock, which is dominated by systemic vasodilation and markedly reduced vascular resistance, leading to refractory hypotension, while the cardiac index (CI) is often preserved or even elevated. The second phenotype is cardiogenic shock secondary to sepsis-induced myocardial dysfunction, characterized by myocardial depression, a significantly reduced cardiac index (typically <2.2 L·min^−1^·m^−2^), and a decreased left ventricular ejection fraction. The third phenotype is right ventricular dysfunction, which is commonly observed in septic patients with concomitant acute respiratory distress syndrome, where increased pulmonary vascular resistance leads to elevated right ventricular afterload and subsequent right heart failure (25).

In patients with septic cardiomyopathy, indications for veno-arterial extracorporeal membrane oxygenation (VA-ECMO) are well-established, including left ventricular ejection fraction (LVEF) ≤ 35%, cardiac index <2.2 L/(min·m^2^), persistent lactate ≥4 mmol/L, and a vasoactive-inotropic score (VIS) ≥ 75 μg/(kg·min) (26). However, the benefit of VA-ECMO in patients with refractory distributive shock and preserved or hyperdynamic left ventricular function remains controversial. Severe systemic inflammation in these patients causes profound vascular leakage and extremely low systemic vascular resistance, making it difficult to maintain effective perfusion even with ECMO, while potentially exacerbating organ perfusion heterogeneity (27).

Extracorporeal cardiopulmonary resuscitation (ECPR) refers to the emergent initiation of VA-ECMO in patients with refractory cardiac arrest unresponsive to conventional CPR, either during ongoing chest compressions or after transient return of spontaneous circulation (ROSC). ECPR temporarily replaces failing cardiac and pulmonary function, preserves perfusion to vital organs, and provides time to identify and treat potentially reversible causes (28). Ideal indications include: witnessed arrest with immediate bystander CPR (no-flow time <5 min), age <70–75 years, initial shockable rhythm or reversible cardiac cause, persistent absence of ROSC after 10–20 min of high-quality ACLS (low-flow time <60 min), and adequate resuscitation quality (ETCO₂ ≥ 10 mmHg) with rapid ECMO deployment capability (29).

The patient experienced intraoperative cardiac arrest during emergency surgery but achieved return of spontaneous circulation (ROSC) with conventional CPR (CCPR) and completed the procedure. Early postoperatively, a second cardiac arrest occurred. Bedside echocardiography demonstrated cardiac standstill with an ejection fraction (EF) < 10%. After 15 min of continuous high-quality CCPR without ROSC, extracorporeal cardiopulmonary resuscitation (ECPR) was initiated, guided by the patient’s relatively young age and potentially reversible etiology. The recurrent circulatory collapse was multifactorial: post-cardiac arrest myocardial stunning led to markedly reduced contractile reserve; emergency surgery and intraoperative hypotension further increased systemic ischemic burden; severe metabolic acidosis impaired myocardial responsiveness to catecholamines and directly suppressed contractility; anesthetic agents exerted additional negative inotropic effects; and abrupt reduction of intra-abdominal pressure following decompression caused a sudden increase in venous return, imposing acute volume load on the stunned myocardium and amplifying ischemia–reperfusion injury, ultimately precipitating a second cardiac arrest.

In this case, after the patient experienced a postoperative cardiac arrest unresponsive to conventional CPR, ECPR in the form of VA-ECMO was promptly initiated. Although high-quality ECPR was delivered, hemodynamics remained unstable, and effective extracorporeal circulation could not be established. This unfavorable outcome was likely due to non-cardiogenic hemorrhagic shock. On one hand, the patient developed severe disseminated intravascular coagulation (DIC) postoperatively, with extensive intra-abdominal and systemic bleeding indicative of hemorrhagic shock. On the other hand, systemic inflammatory response and capillary leak further reduced venous return. Persistent metabolic acidosis and vasoplegia contributed to markedly decreased peripheral vascular resistance and vascular responsiveness. These mechanisms collectively resulted in severely insufficient circulating blood volume, reduced venous return, and impaired vascular tone, leading to low ECMO flow. Consequently, the ECMO system was unable to provide adequate circulatory support to maintain perfusion of vital organs, ultimately resulting in multi-organ hypoperfusion and death.

## Conclusion

For severe acute gastric dilation (AGD), timely and adequate gastrointestinal decompression is the cornerstone of initial management. After nasogastric tube placement, imaging confirmation via CT or bedside radiography is required to ensure correct intragastric positioning and tube patency, with continuous monitoring of decompression efficacy. If decompression is inadequate, replacement with a larger-bore tube (e.g., a lavage tube) should be performed to improve drainage efficiency. For patients in whom conservative management fails or intra-abdominal pressure remains elevated, early surgical intervention is indicated to rapidly evacuate gastric contents and reduce intra-abdominal pressure, thereby preventing catastrophic complications such as circulatory collapse or cardiac arrest. Furthermore, in patients with severe gastric distension causing diaphragmatic elevation, respiratory compromise, or frequent vomiting, early endotracheal intubation is recommended to protect the airway, reduce the risk of aspiration, and improve ventilation. Although extracorporeal membrane oxygenation (ECMO) can provide temporary circulatory support during cardiac arrest, its effectiveness is often limited in cases of vasoplegic distributive septic shock or progressive non-cardiogenic shock, and it may not reverse the underlying pathophysiological derangements.

## Data Availability

The original contributions presented in the study are included in the article/supplementary material, further inquiries can be directed to the corresponding authors.
